# Alcohol Consumption, HDL-Cholesterol and Incidence of Colon and Rectal Cancer: A Prospective Cohort Study Including 250,010 Participants

**DOI:** 10.1093/alcalc/agab007

**Published:** 2021-02-19

**Authors:** Aage Tverdal, Gudrun Høiseth, Per Magnus, Øyvind Næss, Randi Selmer, Gun Peggy Knudsen, Jørg Mørland

**Affiliations:** Norwegian Institute of Public Health, Centre for Fertility and Health, Pb 222 Skøyen, 0213 Oslo, Norway; Norwegian Centre for Addiction Research (SERAF), Institute of Clinical Medicine, University of Oslo, Pb 1171 Blinderen, 0318 Oslo, Norway; Department of Forensic Sciences, Oslo University Hospital, Pb 4950 Nydalen, 0424 Oslo; Center for Psychopharmacology, Diakonhjemmet Hospital, Forskningsveien 13, 0373 Oslo, Norway; Norwegian Institute of Public Health, Centre for Fertility and Health, Pb 222 Skøyen, 0213 Oslo, Norway; Institute of Health and Society, University of Oslo, Pb 1171 Blinderen, 0318 Oslo, Norway; Norwegian Institute of Public Health, Division of Chronic Diseases and Aging, Pb 222 Skøyen, 0213 Oslo, Norway; Norwegian Institute of Public Health, Division of health data and digitalization, Pb 222 Skøyen, 0213 Oslo, Norway; Norwegian Centre for Addiction Research (SERAF), Institute of Clinical Medicine, University of Oslo, Pb 1171 Blinderen, 0318 Oslo, Norway; Norwegian Institute of Public Health, Division of health data and digitalization, Pb 222 Skøyen, 0213 Oslo, Norway

## Abstract

**Aims:**

Alcohol consumption has been linked to colorectal cancer (CRC) and also to the high-density lipoprotein cholesterol level (HDL-C). HDL-C has been associated with the incidence of CRC. The aim of this study was to investigate the association between self-reported alcohol consumption, HDL-C and incidence of CRC, separately for the two sites.

**Methods:**

Altogether, 250,010 participants in Norwegian surveys have been followed-up for an average of 18 years with respect to a first-time outcome of colon or rectal cancer. During follow-up, 3023 and 1439 colon and rectal cancers were registered.

**Results:**

For men, the HR per 1 drink per day was 1.05 with 95% confidence interval (0.98–1.12) for colon and 1.08 (1.02–1.15) for rectal cancer. The corresponding figures for women were 1.03 (0.97–1.10) and 1.05 (1.00–1.10). There was a positive association between alcohol consumption and HDL-C. HDL-C was inversely associated with colon cancer in men (0.74 (0.62–0.89) per 1 mmol/l) and positively associated with rectal cancer, although not statistically significant (1.15 (0.92–1.44). A robust regression that assigned weights to each observation and exclusion of weights ≤ 0.1 increased the HRs per 1 drink per day and decreased the HR per 1 mmol/l for colon cancer. The associations with rectal cancer remained unchanged.

**Conclusion:**

Our results support a positive association between alcohol consumption and colon and rectal cancer, most pronounced for rectal cancer. Considering the positive relation between alcohol consumption and HDL-C, the inverse association between HDL-C and colon cancer in men remains unsettled.

## Teaser

**Short Summary**: This study showed that the relation between alcohol consumption is somewhat stronger for rectal cancer than for colon cancer. Although there is a positive relation between levels of HDL and alcohol consumption, the relation between HDL and colon cancer in men is inverse. This finding warrants further studies.

## INTRODUCTION

Alcohol consumption has been linked to different cancer diseases, and epidemiological studies have specifically reported that the risk of colorectal cancer (CRC) increases with alcohol consumption (Potter, 1996; Moskal *et al.*, 2007; Baena and Salinas, 2015). It is probable that the first metabolite of ethanol, acetaldehyde, which is a well-known carcinogenic factor, contribute to this risk (Seitz *et al.*, 1990; Seitz and Stickel, 2009). Several prospective studies have indicated that the risk increases with increasing consumption levels, but with conflicting data on which levels of alcohol consumption are necessary for increasedrisk.

It has been found that significantly increased risk is seen in individuals reporting to drink two or more drinks per day (Cho *et al.*, 2004; Corrao *et al.*, 2004), and one meta-analysis, including only prospective studies, found a 15% increase in risk of colon or rectal cancer for an increase of 100 g of ethanol intake per week (Moskal *et al.*, 2007). The American Institute for cancer research has summarized that risk of CRC is increased when >30 g of ethanol is consumed per day (www.wcrf.org/dietandcancer/exposures/alcoholic-drinks). Regarding light drinking, the findings are less clear, and one meta-analysis of light alcohol drinking, defined as up to 1 drink per day, did not report any increased risk of CRC (Bagnardi *et al.*, 2013). Another more recent meta-analysis based on 16 studies showed increased risk of CRC in men with light intakes (up to 1 drink/day), when all studies were summarized, but most single studies showed no association. In females, association between light consumption and CRC were non-significant (Choi *et al.*, 2017). This sex difference was not observed in other studies (Cho *et al.*, 2004).

It should be noted that most studies reporting an association between alcohol intake and CRC risk are based on self-reported alcohol intake. As demonstrated in several previous studies, alcohol consumption could be reported inaccurately partly due to lack of sufficiently detailed questions. Underreporting because of several other factors is also possible and this can give biased estimates (Dawson, 2003; Magnus *et al.*, 2011; Klatsky *et al.*, 2014; Livingston and Callinan, 2015). The true consumption level at which significantly increased risk can be expected is therefore unsettled.

Quantitative biomarkers reflecting the degree of ethanol exposure would therefore be useful, but so far, no perfect markers of this type have been discovered. Epidemiological studies have suggested that there is a relation between the magnitude of alcohol intake and the concentration of high-density lipoprotein cholesterol level (HDL-C) in blood at a population level (Berger *et al.*, 2013; Bradley *et al.*, 2016). A significant dose–response between alcohol consumption and HDL-C has also been found in two meta-analyses of experimental studies (Rimm *et al.*, 1999; Brien *et al.*, 2011). It is therefore both experimentally shown that increased alcohol intake lead to increased level of HDL-C and epidemiologically demonstrated that the subjects with the highest alcohol intake show the highest levels of HDL-C. Such increases in HDL-C levels would be assumed to add to individual levels of HDL-C otherwise mainly governed by genetic and other factors. Such other factors are predominantly dietary factors, physical activity and use of lipid lowering drugs (Rimm *et al.*, 1999; Berger *et al.*, 2013). Although this presumably reduces the value of HDL-C measured at one time-point as a perfect quantitative biomarker of alcohol intake, a less subjective indication of alcohol consumption than self-report could be obtained by use of HDL-C concentrations. As far as we know, few studies have used HDL-C levels as support or corrective for self-reported alcohol consumption.

There are also several indications that the level of HDL-C might be related to the incidence of CRC (van Duijnhoven *et al.*, 2011; Pirro *et al.*, 2018; Brantley *et al.*, 2020). A large nested case-control study (van Duijnhoven *et al.*, 2011) found decreasing risk of CRC, in particular colon cancer, with increasing HDL-C levels. The relation between alcohol intake and the risk of CRC therefore appears complex as the effects of alcohol per se might be modified by increases of HDL-C levels induced by alcohol intake. This complexity might be disentangled by a prospective study including both self-reported alcohol consumption, HDL-C levels and the incidence of CRC. To our knowledge, such studies have not been performed sofar.

In the present study, a large cohort of 250,010 men and women recruited in two population based health surveys were followed for a mean period of 18 years in order to investigate the associations between self-reported alcohol consumption and HDL-C levels, between alcohol consumption and incidence of colon and rectal cancer, as well as between HDL-levels and incidence of colon and rectal cancer.

## MATERIALS AND METHODS

The data stems from Norwegian population based health surveys, the year 40 programme (Bjartveit *et al.*, 1991) and the cohort of Norway (Naess *et al.*, 2008).

These surveys consist of information from questionnaires and anthropometric measures, and for the present study the main exposure variable was self-reported intake of alcohol. Altogether, 301,580 men and women aged 20–79 years participated in the surveys. We had information on HDL-C and alcohol consumption for 260,663. Of these 10,653 had missing in one or more of the variables in the multivariate analyses, and excluding these, 250,010 remain as our study population. Furthermore, 389 and 270 of the included participants had a diagnosis of colon or rectal cancer at baseline. We excluded these in the analyses, according to which cancer site we studied.

The participants were asked how many drinks of beer, wine, liquor, specifically for each item, they usually consume during a 14 days period. In total, 213,903 answered these questions. We added the drinks for the three alcohol types, and division by 14 gave drinks per day. For the remaining 46,760 participants we have information about drinking frequency and how many drinks of alcohol were consumed at each occasion. The alcohol frequency question had these pre-set alternatives: 4–7 times/week, 2–3 times/week, once/week, 2–3 times/month, once/month, a few times last year, no alcohol use last year and never used alcohol. These categories were reclassified to 5.5 times/week, 2.5 times/week, 1 time/week, 0.625 times/week, 0.25 times/week, 0.077 times/week, 0 times/week and 0 times/week, respectively. These frequencies were multiplied with number of drinks per occasion, reported by each individual. Division by 7 gave drinks per day. The participants also answered questions about history of cardiovascular disease or diabetes, smoking habits, physical activity and educational level. We measured height, weight and blood pressure at the screening site. A non-fasting blood sample was drawn and serum analyzed for total cholesterol, triglycerides and HDL-C (Naess *et al.*, 2008).

We followed the participants until a colon or rectal cancer diagnosis, death, emigration or 31. Dec 2018, whichever came first, by linkage to the Norwegian Cancer Registry and the Norwegian Cause of Death Registry. Cancer diagnosis was defined according to the international classification of disease (ICD; the Norwegian version of ICD-7), colon cancer was ICD-7153 and rectal cancer was ICD-7154).

During an average follow-up of 18 years, 3023 and 1439 of the study participants were registered with colon and rectal cancer, respectively.

**Table 1:** Age-adjusted mean values and percentages by level of alcohol consumption; men and women 20–79 years

Alcohol (drinks per day)	0	> 0–< 0.5	0.5–< 1	≥ 1
Men				
*N*	23,100	56,232	26,458	15,202
Age	49	47	44	45
Age-adjusted values				
Smoking (%)	23	30	37	43
*N* cigarettes/day	3.9	3.9	5.1	6.8
HDL (mmol/l)	1.15	1.22	1.27	1.32
Cholesterol (mmol/l)	5.73	5.72	5.79	5.83
Triglycerides (mmol/l)	2.11	2.01	2.03	2.05
BMI (kg/m^2^)	26	26	26	26
Height (cm)	178	179	179	179
Systolic b.p. (mmHg)	135	134	134	135
Education (≥ 13 years) (%)	23	31	34	38
Education (0,1,2,3…,7,8)	3.8	4.2	4.3	4.4
Physical active (%)	46	48	49	47
Unmarried (%)	26	23	23	29
Women				
*N*	43,537	66,801	14,661	4019
Age	48	44	43	45
Age-adjusted values				
Smoking (%)	31	35	44	51
*N* cigarettes/day	3.5	3.8	5.3	6.9
HDL (mmol/l)	1.41	1.51	1.58	1.66
Cholesterol (mmol/l)	5.66	5.55	5.50	5.52
Triglycerides (mmol/l)	1.53	1.36	1.31	1.32
BMI (kg/m^2^)	26	25	25	24
Height (cm)	165	165	166	167
Systolic b.p (mmHg)	128	126	125	126
Education (≥ 13 years) (%)	22	32	39	46
Education (0,1,2,3…,7,8)	3.7	4.1	4.3	4.6
Physical active (%)	38	43	47	46
Unmarried (%)	17	17	16	22

We calculated adjusted mean values and proportions by using the ‘adjmean’ procedure in STATA (Station: and 2013). Age was set at 45 years. We used Cox proportional hazards regression to investigate the relationship between alcohol consumption as a risk factor and colon or rectal cancer (event). In this Cox regression, we adjusted for several potential confounders (details reported in each table). Crude and adjusted hazard ratios (HR) for colon and rectal cancer with 95% confidence intervals (CI) are reported for each alcohol consumption group and each HDL-C group. We estimated HR with the proportional hazards model and tested the proportional hazards assumption by a test based on Schoenfeld residuals (StataCorp. 2013). We plotted the association between drinks per day and HDL-C overlaid with a quadratic fit (StataCorp. 2013). We did robust regressions, sex specific, with HDL-C as dependent and age, drinks/day and drinks/day squared as independent variables, where each observation was assigned a weight. We run Cox regressions excluding observations with weight ≤ 0.1 (~1300).

The study was approved by the Regional Committees for Medical and Health Research Ethics (reference number 2009/605 south-east) and The Norwegian Data Protection Authority (reference number 05/01557-3).

## RESULTS

The baseline characteristics of the study population are shown in Table 1. The smoking prevalence, number of cigarettes, the educational length and HDL-C level increased with increasing alcohol consumption (Table 1 and Supplementary Fig. 1).

The mean time between baseline and the diagnosis of colon and rectal cancer was 10.7 and 10.4 years, respectively. The risk of colon cancer in all consumption groups was greater than for non-drinkers, but the HR per 1 drink per day was not significant (Table 2). The exclusion of observations with low weight raised the HR per 1 drink per day, and for men it became statistically significant.

**Table 2:** Number of cases, HR for colon and rectal cancer with 95% CI by alcohol drinks per day; persons 20–79 years

	Colon				Rectum			
	Men							
Drinks/day	Cases	HR^***^	HR^†^	95% CI	Cases	HR^***^	HR^†^	95% CI
0	318	Ref	Ref		151	Ref	Ref	
>0–<0.5	753	1.09	1.14	0.99–1.30	368	1.08	1.11	0.92–1.35
0.5–<1.0	282	1.06	1.13	0.95–1.33	184	1.30	1.32	1.06–1.66
≥ 1.0	179	1.13	1.23	1.01–1.49	117	1.44	1.45	1.12–1.86
Per 1 cat^*^	1532	1.03	1.06	1.00–1.12	820	1.14	1.14	1.06–1.23
Per 1 drink/day	1532	1.03	1.05	0.98–1.12	820	1.09	1.08	1.02–1.15
Per 1 drink/day^**^	1521	1.05	1.08	1.00–1.17	812	1.13	1.08	1.02–1.15
	Women							
0	601	Ref	Ref		212	Ref	Ref	
>0–<0.5	687	1.05	1.05	0.93–1.17	300	1.13	1.18	0.98–1.42
0.5–<1.0	153	1.20	1.18	0.98–1.43	77	1.39	1.47	1.12–1.94
≥ 1.0	50	1.32	1.26	0.93–1.69	30	1.95	2.07	1.39–3.09
Per 1 cat^*^	1491	1.09	1.08	1.00–1.16	619	1.21	1.24	1.11–1.38
Per 1 drink/day	1491	1.05	1.03	0.97–1.10	619	1.06	1.05	1.00–1.10
Per 1 drink/day^**^	1483	1.05	1.04	0.97–1.17	618	1.06	1.05	1.00–1.10

^*^Per 1 category of drinks/day.

^**^Without observations with weight ≤ 0.1 from robust regressions.

^***^Adjusted forage.

^†^Adjusted for age, HDL-cholesterol, total cholesterol, triglycerides, number of cigarettes/day systolic blood pressure, education (1–6 years = 1, 7–9 years = 2, …., 17–18 years = 7, 18+ years = 8, body mass index, height, physical activity (no, yes), unmarried (no,yes).

For rectal cancer there was a clear dose–response relationship with the alcohol groups (Table 2). For both genders the HR per 1 drink per day was significant, and the exclusion of observations did not influence the estimates. Deletion of HDL-C among the covariates only marginally influenced the estimates (data not shown).

**Fig. 1.**
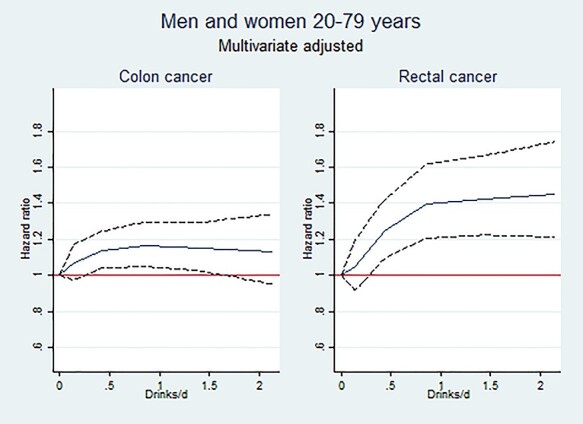
Rectal and colon cancer incidence according to alcohol consumption (men and women combined).

The HR increased up to 1 drink per day for both cancer sites, most steeply for rectal cancer (Fig. 1). Thereafter, there was no further increase. The risk of colon or rectal cancer did not vary between subjects consuming one, two or three types of alcohol (*P* = 0.4 and 0.8 for colon and rectal cancer, respectively).

There was a positive association between alcohol consumption and HDL-C, as seen in Supplementary Fig. 1. Only 0.3% of our study population consumed 3 or more drinks per day. Below this limit, one drink per day was associated 0.07 mmol/l increase in HDL-C for men and 0.10 mmol/l for women.

For colon cancer there is a downward trend for HR across the HDL-C categories in men, but not in women (Table 3). The HR for colon cancer per 1 mmol/l HDL was significantly decreased for men. For rectal cancer the HR per 1 mmol/l HDL-C was not significant. The exclusion of observations with low weight lowered the HR for colon cancer in both men and women.

**Table 3:** Number of cases, HR with 95% CI by HDL cholesterol (HDL-C); persons 20–79 years

	Colon				Rectum			
	Men							
HDL-C mmol/l	Cases	HR^***^	HR^†^	95% CI	Cases	HR^***^	HR^†^	95% CI
<1	326	Ref	Ref		163	Ref	Ref	
1–<1.5	898	0.91	0.95	0.83–1.09	460	0.98	1.02	0.85–1.24
1.5–<2.0	263	0.75	0.82	0.68–0.99	162	1.04	1.12	0.88–1.44
≥2.0	45	0.63	0.71	0.51–0.99	35	1.20	1.30	0.88–1.94
Per 1 cat^*^	1532	0.86	0.90	0.83–0.97	820	1.04	1.08	0.97–1.20
Per 1 mmol/l	1532	0.69	0.74	0.62–0.89	820	1.06	1.15	0.92–1.44
Per 1 mmol/l^**^	1521	0.66	0.70	0.58–0.85	812	1.05	1.14	0.88–1.46
	Women							
<1	106	Ref	Ref		35	Ref	Ref	
1–<1.5	650	0.85	0.91	0.73–1.12	280	1.11	1.21	0.84–1.75
1.5–<2.0	561	0.87	0.96	0.76–1.21	234	1.13	1.30	0.88–1.93
≥ 2.0	174	0.85	0.96	0.73–1.26	70	1.18	1.39	0.88–2.20
Per 1 cat^*^	1491	0.98	1.02	0.94–1.10	619	1.04	1.09	0.97–1.23
Per 1 mmol/l	1491	0.94	1.02	0.87–1.18	619	1.05	1.15	0.91–1.46
Per 1 mmol/l^**^	1483	0.92	1.00	0.85–1.17	618	1.07	1.20	0.94–1.54

^*^Per 1 category of HDL-cholesterol.

^**^Without observations with weight ≤ 0.1 from robust regressions.

^***^Adjusted forage.

^†^Adjusted for age, HDL-cholesterol, total cholesterol, triglycerides, number of cigarettes/day systolic blood pressure, education (1–6 years = 1, 7–9 years = 2, …., 17–18 years = 7, 18+ years = 8, body mass index, height, physical activity (no, yes), unmarried (no,yes).

## DISCUSSION

This study showed a positive association between alcohol consumption and colon and rectal cancer, most prominent for rectal cancer. There was an association between the number of drinks of alcohol reported and HDL-C, but the relation between HDL-C and colon cancer was inverse inmen.

In the present study, we found a somewhat more pronounced association between the alcohol-related risk of rectal cancer compared to colon cancer. This is also found in previous studies (Pedersen *et al.*, 2003), and has been indicated in others (Potter, 1996). One meta-analysis on CRCs found higher relative risks for rectal than for colon cancers among any drinkers and light drinkers, but about the same among moderate and heavy drinkers (>4 drinks per day) (Fedirko *et al.*, 2011). The large meta-analysis by Choi et al found non-significant effects (men and women combined) of very light, light or moderate intake of alcohol on both colon and rectal cancer, but estimates were generally somewhat higher for rectal cancer (Choi *et al.*, 2017). Another meta-analysis concluded that the same association between alcohol intake and risk of cancer was seen for colon and rectum, but estimates were only significant for rectal cancer in subjects drinking 30–45 g of ethanol a day (Cho *et al.*, 2004).

This possible difference between alcohol’s potential to lead to cancer in the colon and rectum could be connected to the primary metabolite of alcohol, acetaldehyde. It is previously shown that the activity of the alcohol dehydrogenase (ADH) isoform present in the large intestine (ADH class III), is higher in the rectal mucosa compared with the remaining colon (Seitz *et al.*, 1996; Chiang *et al.*, 2012). This could lead to higher local concentrations of carcinogenic acetaldehyde (Seitz and Stickel, 2009), and thereby higher risk of cancer development. It should however be noted that another low dose ethanol study did not find the same difference between ADH activity in the distal and proximal colonic mucosa, but rectal mucosa was not studied (Yin *et al.*, 1994). Earlier studies have also indicated that cancers arising in the colon or rectum might have different etiologies depending on site (van Duijnhoven *et al.*, 2011) giving indirect support, but no explanation to our finding of different associations of colon and rectal cancer with alcohol consumption.

The present study also adds knowledge to the question of which consumption level of alcohol is necessary for increased risk of CRC to be present. From the HRs seen in Table 2, 0.5–1 drink of alcohol a day seem to be related to ~15% increased risk of colon cancer and ~40% increased risk of rectal cancer. If defining one drink of alcohol as one unit of alcohol (12 g of pure ethanol), this level where increased risk is seen is somewhat lower than what we have learned from some previous meta-analyses (Cho *et al.*, 2004; Moskal *et al.*, 2007), showing increased risk in those consuming more than two drinks a day or 100 g a week, respectively. It is also lower than the conclusion from the American Institute of Cancer Research (www.wcrf.org/dietandcancer/exposures/alcoholic-drinks), stating that increased risk is seen in those consuming >30 g of ethanol a day. Other reviews found 60% increased risk of CRC among the heaviest drinkers (Huxley *et al.*, 2009) and increased risk of CRC in individuals consuming 50 g ethanol a day (Seitz and Stickel, 2007). On the other hand, our consumption estimate was in accordance with results from men in the previous low alcohol dose meta-analysis (Choi *et al.*, 2017), whereas the study of Bagnardi et al (Bagnardi *et al.*, 2013) did not find increased risk of CRC in those drinking up to 1 drink per day. A previous study also found evidence of a J-shaped profile with decreased risk of CRC in the light drinkers (McNabb *et al.*, 2019), this is not supported by the present investigation.

The problem of inaccurate reporting may be an issue when information of alcohol consumption is based on self-report. A ‘drink’ of alcohol is a non-precise expression, and some participants might think a ‘drink’ of beer represents e.g. 0.5 l (containing ~18 g), not exactly one unit of ethanol (12 g). This may have biased the results in the direction of unrealistically low doses of alcohol being associated with risk of CRC. According to calculations from Statistics Norway and a publication of distributions of alcohol consumption in the USA, 20% of the general population should be expected to drink on average 4.5 units per day (Greenfield and Rogers, 1999; Magnus *et al.*, 2011). In our study, <10% reported to drink >1 drink of alcohol a day, and a combination of different definition of ‘drinks’ and under-reporting is therefore indicated. Recruitment bias with less inclusion of heavy drinkers in our study as well as in other population-based studies is likely and might be another explanation.

According to a meta-analysis of previous experimental studies investigating changes in HDL-C after different alcohol intakes, an increase in HDL-C concentration of 8.3% is seen in those consuming 30 g of ethanol a day (0.0035 mmol/l per gram pure ethanol or 0.042 mmol/l per unit consumed per day), compared to abstainers (Rimm *et al.*, 1999). Very similar results were obtained by Brien *et al.* (2011), showing a mean increase in HDL-C of 0.103 mmol/l in those drinking 30–60 g/day (Rimm *et al.*, 1999; Brien *et al.*, 2011). The use of HDL-C as a scaled biomarker for average alcohol consumption has been proposed (Berger *et al.*, 2013; Bradley *et al.*, 2016), and a large number of studies indicate a strong relation between HDL-C and alcohol consumption on a population level, although it should be noted that HDL-C is affected also by many other factors (Hines *et al.*, 2001; Tolstrup *et al.*, 2009). In the present study, the results indicate that increasing the consumption with one drink of ethanol was accompanied by 0.07 mmol/l increase in HDL-C in men and 0.10 mmol/l in women. This indicates that reported consumption of one ‘drink’ corresponds to a true consumption of 20–30 g pure ethanol. The HDL-C measurements indicated that inaccurate reporting was somewhat less pronounced than previously shown (Magnus *et al.*, 2011), possibly explained by the different way of measuring alcohol consumption.

As we find a close relation between alcohol consumption and HDL-C, we would also expect the same relation between HDL-C and cancer as between alcohol consumption and cancer. However, we found that increasing HDL-C was negatively associated with colon cancer in men. These findings are supported by a previous large case-control study reporting that HDL-C was significantly inversely associated with colon cancer risk while this association was absent for rectal cancer (van Duijnhoven *et al.*, 2011). Other studies have also indicated that HDL-C is generally protective for the development of colorectal and other cancer forms, which could e.g. be related to anti-inflammatory effects of HDL-particles (Schoen *et al.*, 1999; Ahmed *et al.*, 2006; Kajani *et al.*, 2018; Hao *et al.*, 2019). The magnitude of the relationship between alcohol and colon or rectal cancer was the same when excluding HDL-C as a covariate in the analyses, indicating that HDL-C is not a mediator of alcohol’s effects.

We found no clear differences between men and women in alcohol-related risk of colon and rectal cancer in the present study (Tables 2 and 3). A sex difference has been indicated previously (Fedirko *et al.*, 2011; Choi *et al.*, 2017). On the other hand, the relation between HDL-C and colon cancer seemed to be gender related, but we have no explanation forthis.

Strengths of the present study include the very large material including both sexes, to the best of our knowledge larger than previous comparable studies. In addition, we have a long period of complete follow-up, detailed information from the questionnaires and anthropometric measures and data on cancer incidence from national registries.

Limitations of the present study are alcohol consumption report and HDL-C measurement only at a single time-point. It should also be especially noted that confounding variables in the relation between alcohol intake and CRC are numerous and only partly corrected for in the present study. For instance, we lack information about different dietary aspects (Giovannucci *et al.*, 1995), like folate and intake of red meat, type of bacteria present in the gut (Jokelainen and Siitonen, 1996), as well as ADH genotype (Homann *et al.*, 2009). We would especially like to comment on the ADH1C genotype, as a subpopulation of the present material (742 CRC cases and 1312 age matched controls) was also genotyed for the two single nucleotide ADH1C variants (rs1693482 and rs698) of this enzyme (data not shown). The results showed that the effect of alcohol on hazard for CRC did not differ when we stratified by ADH1C genotype. We therefore found no evidence for an additional effect of ADH1C genotype on the relationship between alcohol consumption and CRC. It should however be mentioned that this data could be underpowered and could not be used to draw final conclusions. Finally, although some measurements of each individual’s physical condition (like tri glycerides, total cholesterol, blood pressure, physical activity and obesity) are included in the analyses, there are still some important aspects of a metabolic syndrome that could affect the relations, like diabetic conditions.

In conclusion, the present study found an increased risk of colon and rectal cancer with increasing alcohol consumption, and this was most pronounced for rectal cancer. There was a positive relation between alcohol consumption and HDL-C. The inverse relation between HDL-C and colon cancer suggests a specific modifying effect of HDL-C on cancer development. This finding warrants further studies.

## AUTHORS’ CONTRIBUTIONS

AT performed the statistical analyses and participated in preparing the manuscript. GH drafted the manuscript and coordinated all feedbacks from co-authors. PM, ØN and GPK participated in the design of the manuscript. RS was the leader of the epidemiological project and participated in the design of the manuscript. JM supervised the project and participated in the design and preparation of the manuscript. All authors accepted the last version of the manuscript.

## ETHICAL APPROVAL

All procedures performed in studies involving human participants were in accordance with the ethical standards of the institutional and/or national research committee and with the 1964 Helsinki declaration and its later amendments or comparable ethical standards. The study was approved by the Regional Committees for Medical and Health Research Ethics (reference number 2009/605 south-east) and The Norwegian Data Protection Authority (reference number 05/01557-3).

## CONSENT TO PARTICIPATE

All participants signed informed consent to participate in the study.

## CONSENT FOR PUBLICATION

All participants signed informed consent.

## DATA AVAILABILITY

The results in this study are based on de-identified data from human research participants. For more information, please contact the Norwegian National Institute of Public health, Division of Health Data and Digitalisation (lhu@fhi.no). The reason why the data are not made publicly available in a public repository or as supporting information is because of local legal restrictions as well as ethical restrictions related to privacy. The participants have not consented to their data becoming publicly available and they might lose control of their data, such as their right to have their data deleted. The data underlying this article will be shared on reasonable request to the corresponding author.

## FUNDING

No funding was obtained for the authorship of this manuscript.

## CONFLICT OF INTEREST

The authors declare no conflict of interest.

## Supplementary Material

Supplementary_figure_1_agab007
