# Supplementary figures and images for: Alcohol Consumption, HDL-Cholesterol and Incidence of Colon and Rectal Cancer: A Prospective Cohort Study Including 250,010 Participants
Source: Alcohol Alcohol. 2021 Feb 19;56(6):718–25. doi: 10.1093/alcalc/agab007 (PMC8557640; doi:10.1093/alcalc/agab007)

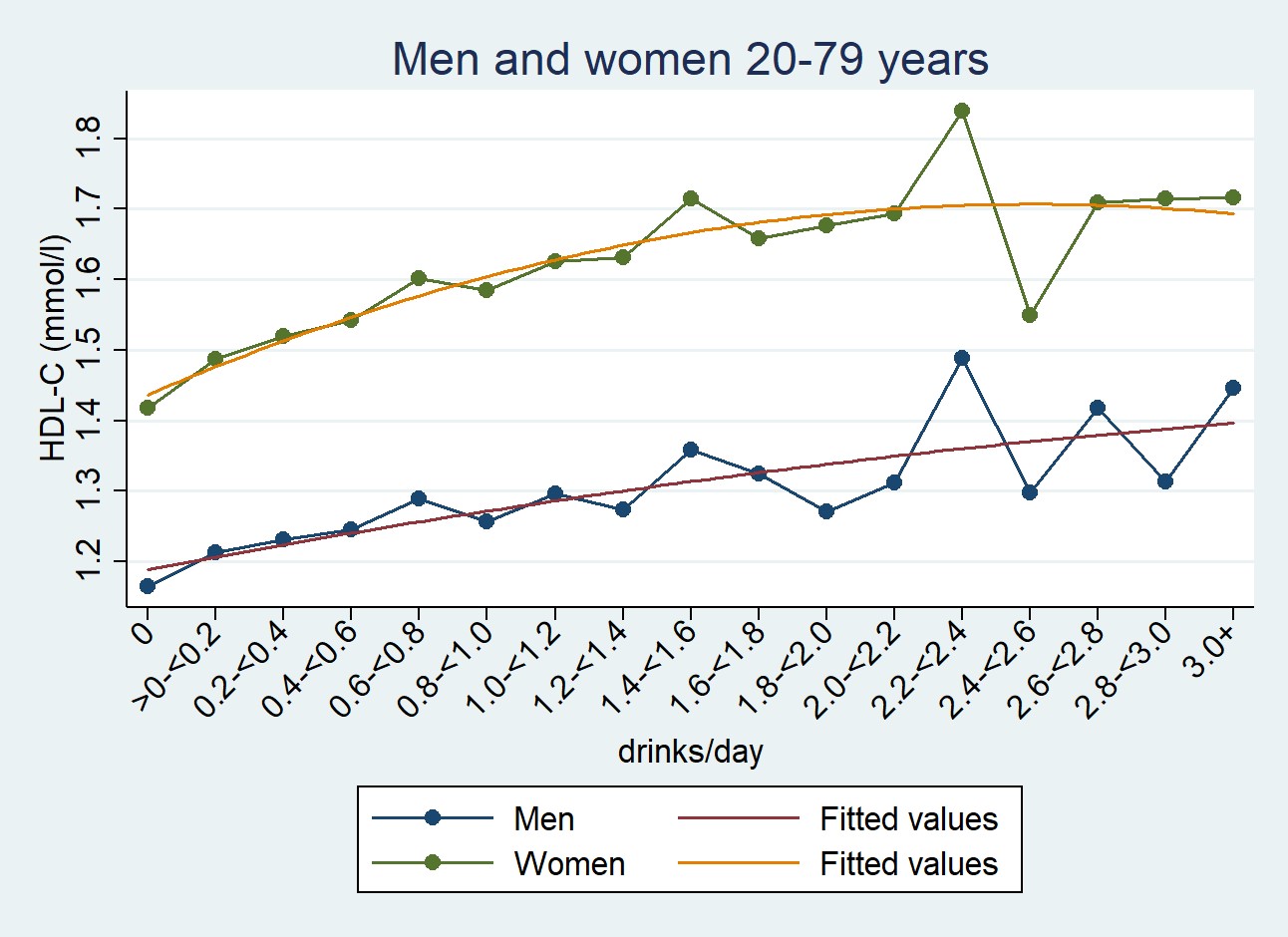

Supplement: Supplementary_figure_1_agab007 [file supplementary_figure_1_agab007.jpeg]
